# Color Modulates Feature Integration

**DOI:** 10.3389/fpsyg.2021.680558

**Published:** 2021-06-11

**Authors:** Harpreet Saini, Heather Jordan, Mazyar Fallah

**Affiliations:** ^1^Department of Biology, York University, Toronto, ON, Canada; ^2^Centre for Vision Research, York University, Toronto, ON, Canada; ^3^Vision: Science to Application (VISTA), York University, Toronto, ON, Canada; ^4^School of Kinesiology and Health Science, York University, Toronto, ON, Canada; ^5^Department of Human Health and Nutritional Sciences, College of Biological Science, University of Guelph, Guelph, ON, Canada

**Keywords:** attention, bayesian perception, visual perception, feature integration, flash-jump effect

## Abstract

Bayesian models of object recognition propose the resolution of ambiguity through probabilistic integration of prior experience with available sensory information. Color, even when task-irrelevant, has been shown to modulate high-level cognitive control tasks. However, it remains unclear how color modulations affect lower-level perceptual processing. We investigated whether color affects feature integration using the flash-jump illusion. This illusion occurs when an apparent motion stimulus, a rectangular bar appearing at different locations along a motion trajectory, changes color at a single position. Observers misperceive this color change as occurring farther along the trajectory of motion. This mislocalization error is proposed to be produced by a Bayesian perceptual framework dependent on responses in area V4. Our results demonstrated that the color of the flash modulated the magnitude of the flash-jump illusion such that participants reported less of a shift, i.e., a more veridical flash location, for both red and blue flashes, as compared to green and yellow. Our findings extend color-dependent modulation effects found in higher-order executive functions into lower-level Bayesian perceptual processes. Our results also support the theory that feature integration is a Bayesian process. In this framework, color modulations play an inherent and automatic role as different colors have different weights in Bayesian perceptual processing.

## Introduction

Our ability to recognize and interact with objects relies heavily on visual perception. However, images of real-world stimuli can be highly complex and ambiguous. Despite ambiguous, incomplete or noisy input, the human visual system is able to perceive objects and object properties with great accuracy. Helmholtz (1867) theorized that the visual system makes unconscious deductions or “inferences” about object and scene properties to resolve this ambiguity, resulting in accurate perception. In recent decades, Helmholtz's theory of unconscious inference has been formalized into models of Bayesian perception. Bayesian models of visual perception suggest that the resolution of ambiguity occurs through probabilistic integration of prior experience or knowledge (priors) with available sensory information (likelihood), giving rise to a probability distribution of the object property in question. With noisier sensory input, there is increased reliance on prior knowledge (Kersten et al., 2004).

Color is an important source of information in a wide variety of daily tasks ranging from object perception to response control. The specific color of an object aids in object recognition, such as the color of an apple distinguishing between varieties. When driving, a red traffic light alerts the driver to stop the vehicle. However, recent studies have shown task-irrelevant color can also intrinsically modulate cognitive functions such as attentional selection (Tchernikov and Fallah, 2010), visual search (Lindsey et al., 2010), and response inhibition; (Blizzard et al., 2017). Tchernikov and Fallah (2010) demonstrated that smooth pursuit target selection was dependent on an intrinsic attentional hierarchy of red (strongest), followed by green, then yellow, and blue (weakest) when saccading to two superimposed random-dot kinetograms. In the absence of any task demand to follow a particular color, red was automatically selected and pursued over other colors; green was marginally selected and pursued over yellow and blue, and yellow was selected and pursued over blue; whereas, blue was not selected over any of the other colors. Similarly, Lindsey et al. (2010) reported that red targets were faster to find in a visual search task compared to other colored targets. Blizzard et al. (2017) reported that response inhibition was facilitated by red more than green stop signals in a stop-signal task (SST). Electrophysiological studies have also provided support for color modulations of executive functions. In a visual search task, red and blue targets evoked earlier N2pc waveforms compared to other colors, suggesting that the deployment of attention may occur faster for red and blue (Fortier-Gauthier et al., 2013; Pomerleau et al., 2014). In addition, Racey et al. (2019) performed an fMRI study which demonstrated that the posterior midline cortex (PMC), which is involved in preferences and value judgements of stimuli (Kable and Glimcher, 2007; Grueschow et al., 2015), was modulated by color features, even if color was irrelevant to the orientation judgment task, which supports different colors having different values or weights. Taken together, these investigations suggest that the color of visual input can intrinsically modulate attentional and cognitive processes, driving differences in later stage decision making processes which ultimately modulate behavioral control. We propose that this color dependent modulation of visual processing can be explained by Bayesian models of perception. In a Bayesian framework, the relative strength of a given color would vary the weight of the priors, biasing the joint probability distribution, and potentially produce different behavioral outcomes based on color.

In the present study, we sought to investigate whether such color-dependent modulations are dependent on Bayesian perceptual processes in the visual processing streams. We focused on feature integration using the flash-jump effect, a visual illusion of color and motion integration first described by Cai and Schlag (2001). Sundberg et al. (2006) found neural correlates of the flash jump effect in area V4 and propose that a Bayesian framework for motion and flash integration underlies this illusion. In the flash jump illusion, a moving stimulus such as a rectangular bar changes color at a single position along its trajectory. Observers perceive the flash as occurring farther along the trajectory of motion, than at its veridical position, thus mislocalizing the flash to a later occurring bar in the sequence. This is a mislocalization of feature integration between color and motion information. If the flashed element is the last element in the sequence (terminating condition), the flash location is perceived veridically. Sundberg et al. (2006) recorded the responses of color selective V4 neurons as monkeys viewed the flash jump illusion. In the classic continuing motion condition, the receptive fields of V4 neurons that were selective for the color of the flash shifted along the trajectory of motion, which matched psychophysical data in humans viewing the same stimuli. Interestingly, when the flash terminated the motion sequence, the retinotopic shift in V4 persisted, supporting a mislocalization of the color and motion feature integration. However, humans reporting the flash location in the terminating condition did not mislocalize the flash. Previous studies have used Bayesian frameworks to account different aspects of real and illusory motion processing (Jacobs, 1999; Weiss et al., 2002; Lisi and Cavanagh, 2015, Gershman et al., 2016; Hui et al., 2020; Yang et al., 2021). Building on those studies, Sundberg et al. (2006) suggest that this motion dependent mislocalization in the continuing but not terminating condition is consistent with a Bayesian model of sensory integration (see: Knill, 2007; Vilares and Kording, 2011), supported by the retinotopic mislocalization in area V4 neurons. The position of the color flash in area V4 and the positions of the moving bars in motion areas such as MT are integrated together to give a joint probability estimate of the color flash in relation to the motion sequence. Sundberg et al. (2006) posit that the presence or absence of a shift, observed in the continuing and terminated conditions is a result of this integration, where the representation of the flash (V4 responses) biases the joint estimation in the continuing condition resulting in large perceptual shifts. However, in the terminating condition, there are no representations for any further bar positions and thus the joint estimation results in a veridical representation of the flash location.

In the present study, we investigated whether varying the isoluminant color (red, green, yellow, or blue) of the flash affected its mislocalization. We hypothesized that in a Bayesian framework, colors associated with stronger attentional capture, i.e., red (Tchernikov and Fallah, 2010; Fortier-Gauthier et al., 2013), would have stronger weights resulting in a decrease in the mislocalization error of the flash. Furthermore, the pattern of color shifts would distinguish which underlying mechanism drives perception: the intrinsic color hierarchy (Tchernikov and Fallah, 2010) which requires color space representation, or “pop-out” visual search color advantages for red and blue (Pomerleau et al., 2014), which are likely dependent on color opponency effects.

## Methods

### Participants

Twenty-four undergraduate students (17 females and 7 males; 18–43 years) completed the study for course credit. The study was approved by the York University Human Participants Research Committee. All participants were naïve to the purpose of the study, had normal or corrected-to-normal acuity and normal color vision (Ishihara, 2006). All participants gave written informed consent prior to participation.

### Paradigm

Participants were seated in a dimly lit room with their heads resting on a chin rest 84 cm from an 18” CRT monitor (60 Hz refresh, 1,024 × 768). Eye position was tracked using an infrared eye-tracking system (ISCAN, Inc. ETL-400), and experimental control was handled by Presentation (Neurobehavioral Systems). The background was dark gray (CIE *x* = 0.35, *y* = 0.56; 0.365 cd/m^2^) with a light gray fixation cross (0.34° by 0.34° CIE *x* = 10.83, *y* =16.25; 11.93 cd/m^2^) at the center of the display.

**Figure 1:**
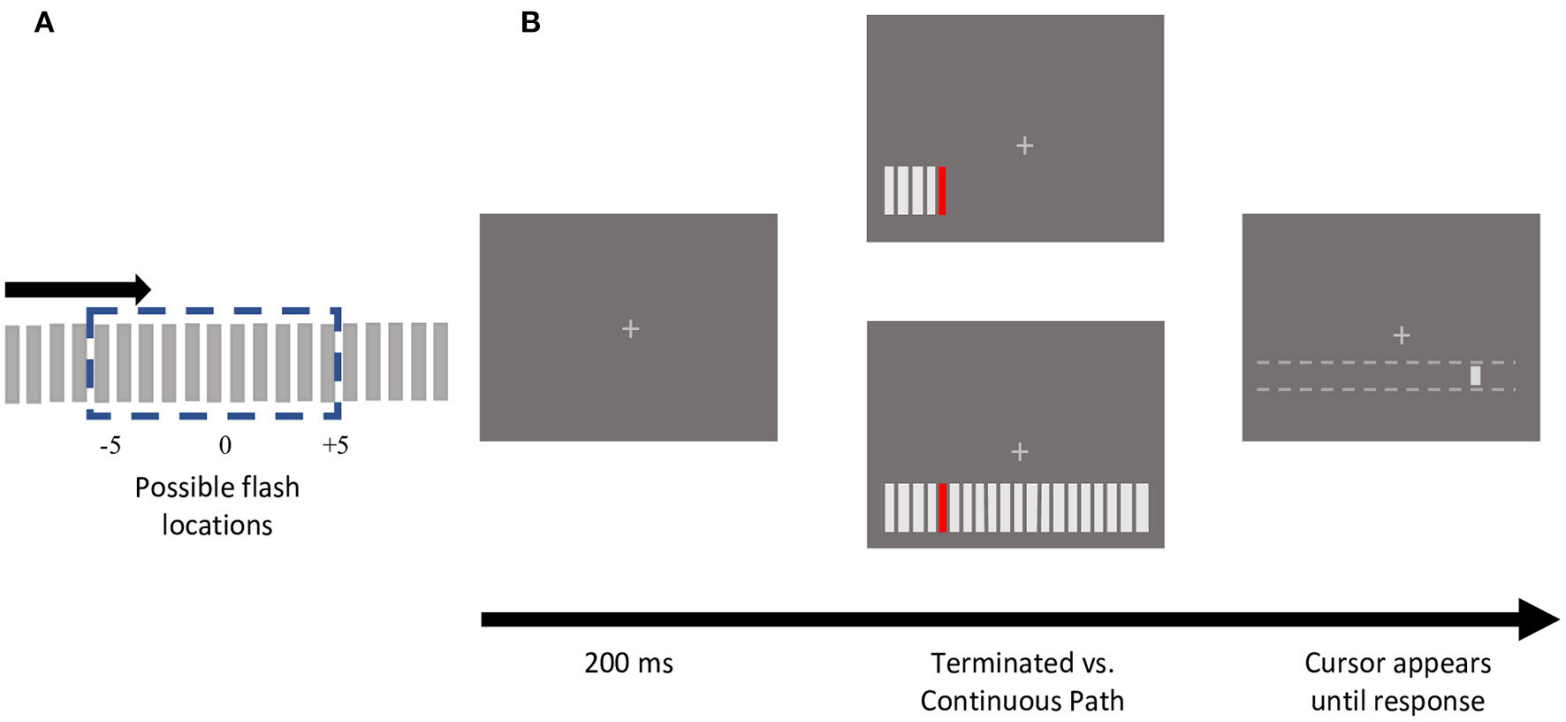
Stimulus schematic and sample trial presentation. **(A)** An apparent motion sequence comprised of 20 gray, rectangular bars, moved either in a leftward (not depicted) or rightward direction. One of the central 11 positions was presented in one of 4 isoluminant colors (red, green, yellow, and blue). **(B)** Experimental protocol of trials depicting sample terminating vs. continuing motion path trials. For each motion path (terminating or continuing), each of the 11 central bar positions were tested, using both directions, and all 4 colors. Participants reported the colored flash location by clicking with a mouse, that could only move horizontally along the motion path. Note that the dotted white lines did not appear on the screen.

The stimulus consisted of an array of 20 bars (3.3° by 0.3°), spaced 0.8° apart, presented 4° below the fixation cross (Figure 1). Apparent motion sequences were produced by bars sequentially appearing for 2 frames, with 2 frames of blank time in between, moving either leftwards or rightwards. The inducing bars were light gray, matching the fixation cross (CIE *x* =10.83, *y* =16.25; 11.93 cd/m^2^). The target, a colored bar selected from one of four photometrically isoluminant colors (12 cd/m^2^; Red *x* = 27.57, *y* = 1.804; Green *x* = 6.007, *y* = 2.516; Yellow *x* = 12.24, *y* = 2.379; Blue *x* = 27.08, *y* = 139.3) appeared at one of the 11 central positions (Figure 1A). Consistent with prior studies of color modulations of visual or cognitive processing, photometric isoluminance was necessary to determine the modulatory effects of individual colors as, perceptual isoluminance would have incorporated these effects (as well as others) into the resulting perceptual luminance for each color.

In the terminating condition, once the colored bar appeared, the motion sequence was terminated such that this flashed element was the last bar presented (Figure 1B). In the continuing condition, the bar appeared at all 20 positions (Figure 1B). If the participant broke fixation during stimulus presentation, the trial was terminated with an auditory warning and shuffled back into the same block.

At the end of the apparent motion sequence, if fixation was maintained successfully, a light gray cursor bar (0.55° by 0.3°) (CIE *x* =10.83, *y* =16.25; 11.93 cd/m^2^) appeared 4° below fixation. The mouse could shift the cursor horizontally, but not vertically. The cursor's initial location was randomized along the length of the stimulus array (−12.29 to +12.29°). Using the mouse, participants reported the position of the colored target flash by moving the cursor to the perceived location and clicking. Participants did not receive any feedback on the accuracy of their responses.

Participants completed 4 blocks of trials with eye-tracking calibration at the beginning of each block, and as required during the experiment. In each block, participants completed 176 trials, one for each of the 11 target flash locations crossed with each of the 4 target colors, 2 motion paths (terminating vs. continuing), and both motion directions (leftward vs. rightward).

### Data Analysis

**Figure 2:**
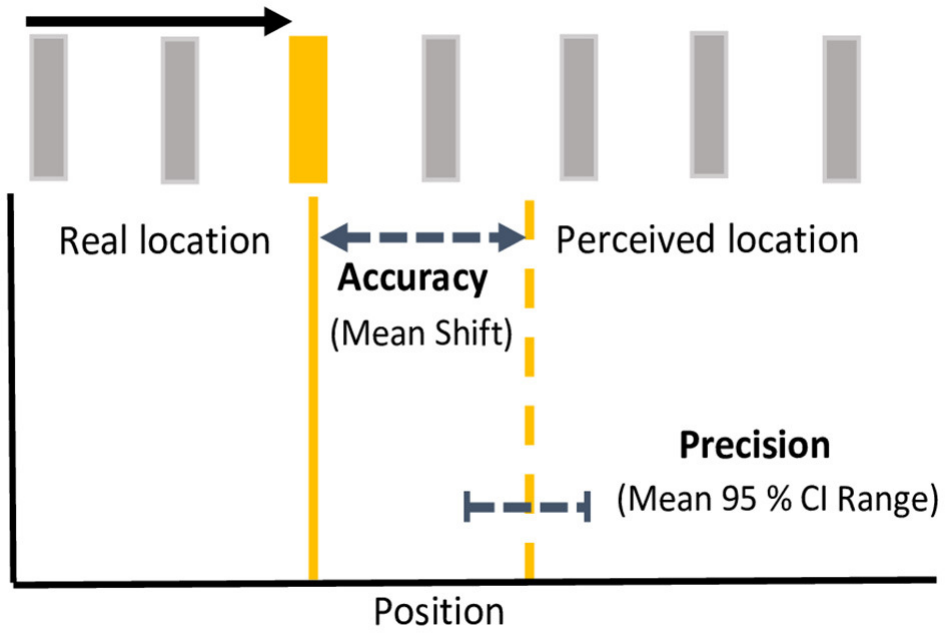
Analytical metrics. For each motion path and flash color, response accuracy was measured as the mean shift between the real location (solid line) and the perceived location (dashed line) of the flash. Response precision was measured as the mean range (upper-lower bound) of 95% confidence intervals for the perceived shifts. Accuracy and precision depicted here is based on mean metrics for yellow flashes in the continuing condition. Note that spacing between bars is exaggerated for illustration.

To examine the effects of color on the magnitude of the flash jump effect, the horizontal distance (dva) of between the location the target flash and the perceived location was computed such that a positive discrepancy was forward, and a negative discrepancy backward along the direction of apparent motion. The discrepancy between target location and response was collapsed separately for terminating vs. continuing conditions across all 11 possible flash positions and both directions of motion. Population means were derived from median shifts in perceived flash locations for each participant. One-sample, one-tailed *t*-tests, corrected for multiple comparisons using an incremental application of the Bonferroni method (Benjamini and Hochberg, 1995), were used to investigate whether mean flash location shifts were significantly mislocalized forward from zero. To investigate color differences in the precision of localization responses, we computed 95% confidence intervals (CI) of the distribution of position responses (horizontal position) for each motion path and flash color, by participant, as has been done previously in eye movement and reach targeting studies (Henriques et al., 2003; Khan et al., 2005; Ren et al., 2006; Blohm and Crawford, 2007). The upper bound was subtracted from the lower bound of the 95% CI to give an estimated width or range as a metric of response precision. See Figure 2 for a schematic depiction of analytical metrics. Mean differences in mislocalization shifts and precision metrics were analyzed separately using 4 flash color (red, green, yellow, blue) X 2 motion path (terminating, continuing) repeated measures ANOVAs. Benjamini and Hochberg (1995) corrections were applied to *post-hoc* tests to control for multiple comparisons.

## Results

**Figure 3:**
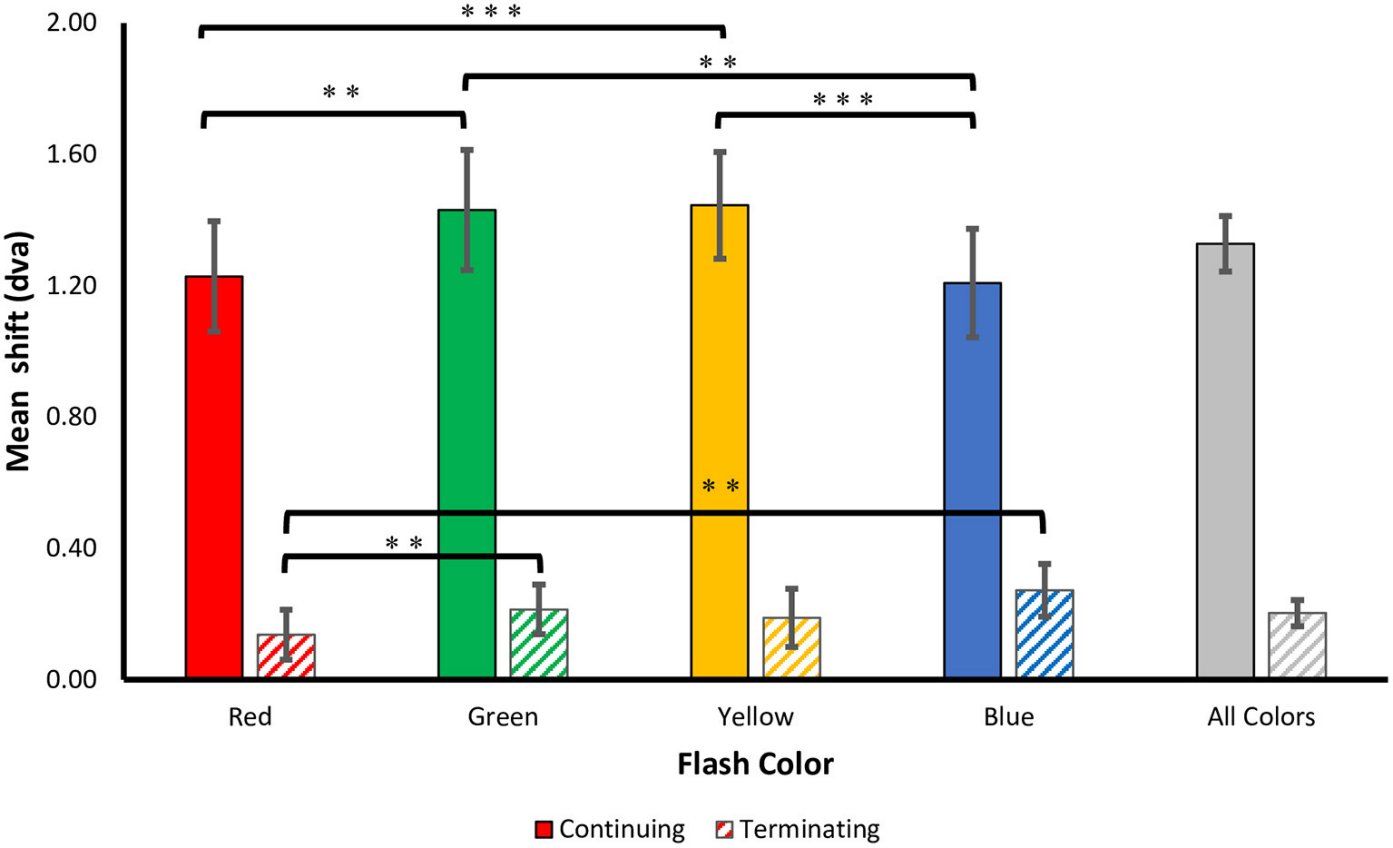
Perceived shifts. The mean ± SEM shift (dva) in the perceived flash location for both terminating and continuing motion paths. Positive values correspond to a forward shift along the direction of motion. Corrected **p < 0.01, ***p < 0.001.

The mean difference between the veridical and participants' perceived locations were calculated for each flash color in the continuing and terminating conditions, listed in Table 1 and shown in Figure 3. One-sample one-tailed *t*-tests showed that the reported flash locations were consistently and significantly mislocalized forward along the path of motion for all colors in the continuing path condition (all large effect sizes by Cohen's d) as well as the terminating path condition (all corrected *p*'s < 0.05, red and yellow: small effect sizes, green and blue: medium effect sizes).

**Table 1:** Mean shift (dva) in flash location and significance values (corrected *p*-value, Cohen's d) for each motion path.

	**Flash color**	**Mean shift (dva)**	***T*-test Sig. (1-tailed)**	**Cohen's d**
Continuing (1.33 dva)	Red	1.23	<0.001	1.490
	Green	1.43	<0.001	1.595
	Yellow	1.45	<0.001	1.809
	Blue	1.21	<0.001	1.493
Terminating (0.2 dva)	Red	0.14	0.043	0.366
	Green	0.21	0.01	0.575
	Yellow	0.19	0.029	0.434
	Blue	0.27	0.006	0.690

To determine whether the magnitudes of the flash-jump mislocalizations were modulated by color, a 4 (Flash Color: red, green, blue, yellow) X 2 (Motion Path: terminating, continuing) repeated measures ANOVA was carried out.

Consistent with prior studies of the flash-jump illusion, we found a significant main effect for motion path [*F*_(1, 69)_ = 60.504, *p* < 0.001, $\eta_{p}^{2}$ = 0.725], with a much smaller mislocalization in the terminating [*M (SD)* = 0.20 (0.39)] compared to the continuing [*M (SD)* = 1.33 (0.83)] motion condition. Supporting our hypothesis, there was a main effect for flash color [*F*_(3, 69)_ = 7.709, *p* < 0.001, $\eta_{p}^{2}=$ 0.251], showing that the isoluminant color of the flash affected the magnitude of the jump. There was a significant interaction between motion path and flash color [*F*_(3, 69)_ = 9.731, *p* < 0.001, $\eta_{p}^{2}=$ 0.297], resulting from a stronger color modulation on flash localization in the continuing motion path than in the terminating motion path.

To investigate this further, separate one-way ANOVAs were carried out on each motion path condition. In the continuing path condition, there was again a significant main effect of flash color, *F*_(3, 69)_ = 11.000, *p* < 0.001, $\eta_{p\;}^{2}=$ 0.324. Mislocalization was smaller for red [*M (SD)* = 1.23 (0.82)] and blue [*M (SD)*= 1.21 (0.81)], than for green [*M (SD)* = 1.43 (0.90)] and yellow [*M (SD)* = 1.45 (0.80)] flashes. Planned pairwise comparisons with Benjamini-Hochberg corrections show no significant differences between red and blue (corrected *p* = 0.86) or green and yellow (corrected *p* = 0.822), but all other color differences were significant (corrected *p*'s < 0.003) (Figure 3). This pattern of results was robust across individuals, as 19 out of 24 participants produced smaller shifts for red compared to green flashes, and 21 out of 24 participants produced smaller shifts for blue compared to yellow flashes.

**Figure 4:**
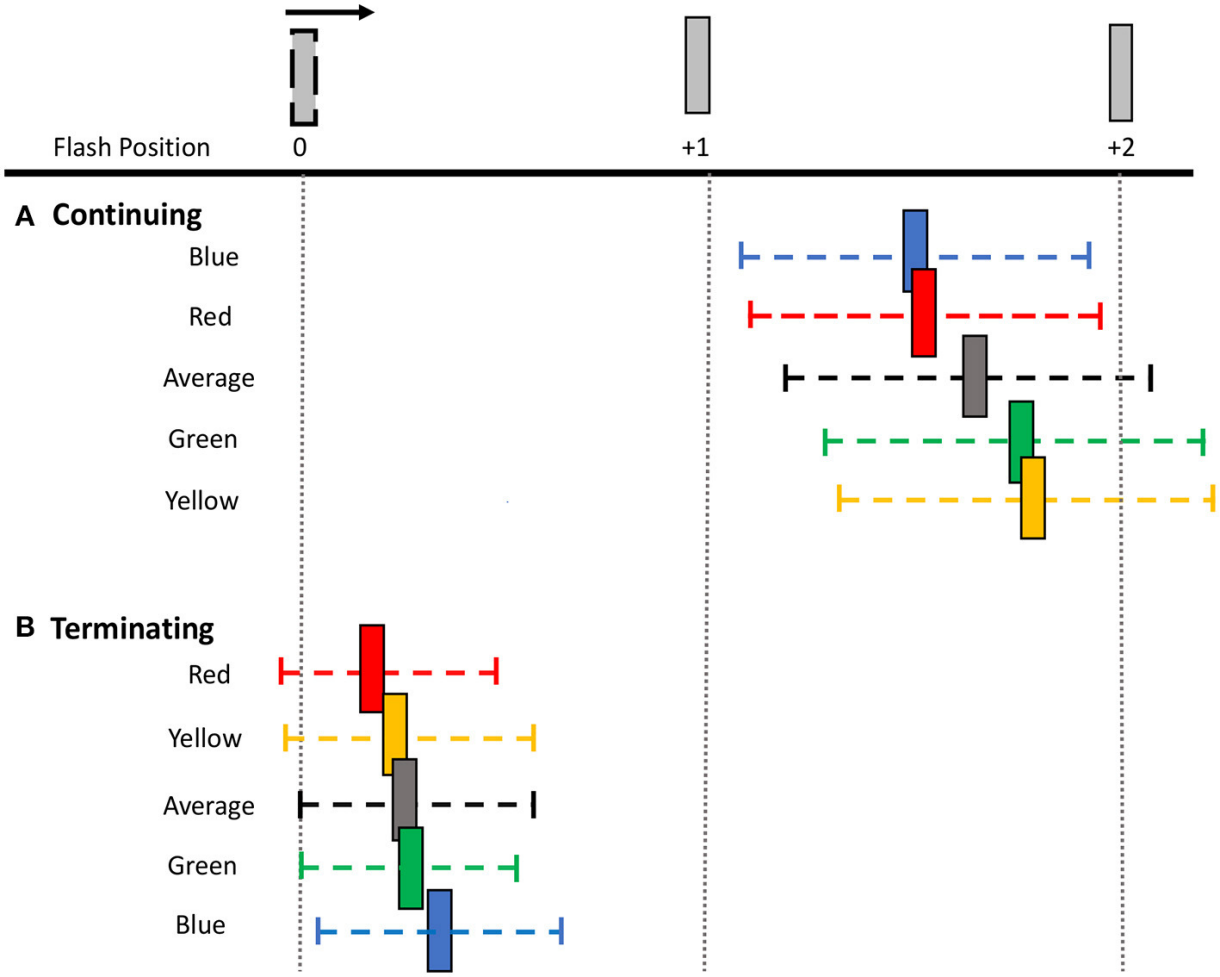
Perceived shifts in flash location relative to bar positions. The mean shift (colored bar) and mean 95% CI range (colored dashed line) for each flash color and overall average for both **(A)** continuing and **(B)** terminating motion paths. Note that the flash appears at bar position 0 and the apparent motion sequence moves rightwards. Spacing between bars is exaggerated for illustration purposes.

In the terminating path condition, where the overall flash-jump effect was small [*M (SD)* = 0.20 (0.39)], there was still a significant main effect of flash color, [*F*_(3, 69)_ = 4.190, *p* = 0.009, $\eta_{p\;}^{2}=$ 0.154]. The smallest shift was observed for red [*M (SD)* = 0.14 (0.37)], followed by yellow [*M (SD)* = 0.19 (0.43)], green [*M (SD)* = 0.21 (0.37)], and blue [*M (SD)* = 0.27 (0.39)] flashes. Planned pairwise comparisons show a significant difference between red and both green (corrected *p* = 0.009) and blue flashes (corrected *p* = 0.009). None of the other contrasts were significantly different (all corrected *p*'s > 0.05) (Figure 3). Therefore, the pattern of results across the four colors differed between the continuing and terminating conditions (see Figure 4 for a summary of color differences).

**Figure 5:**
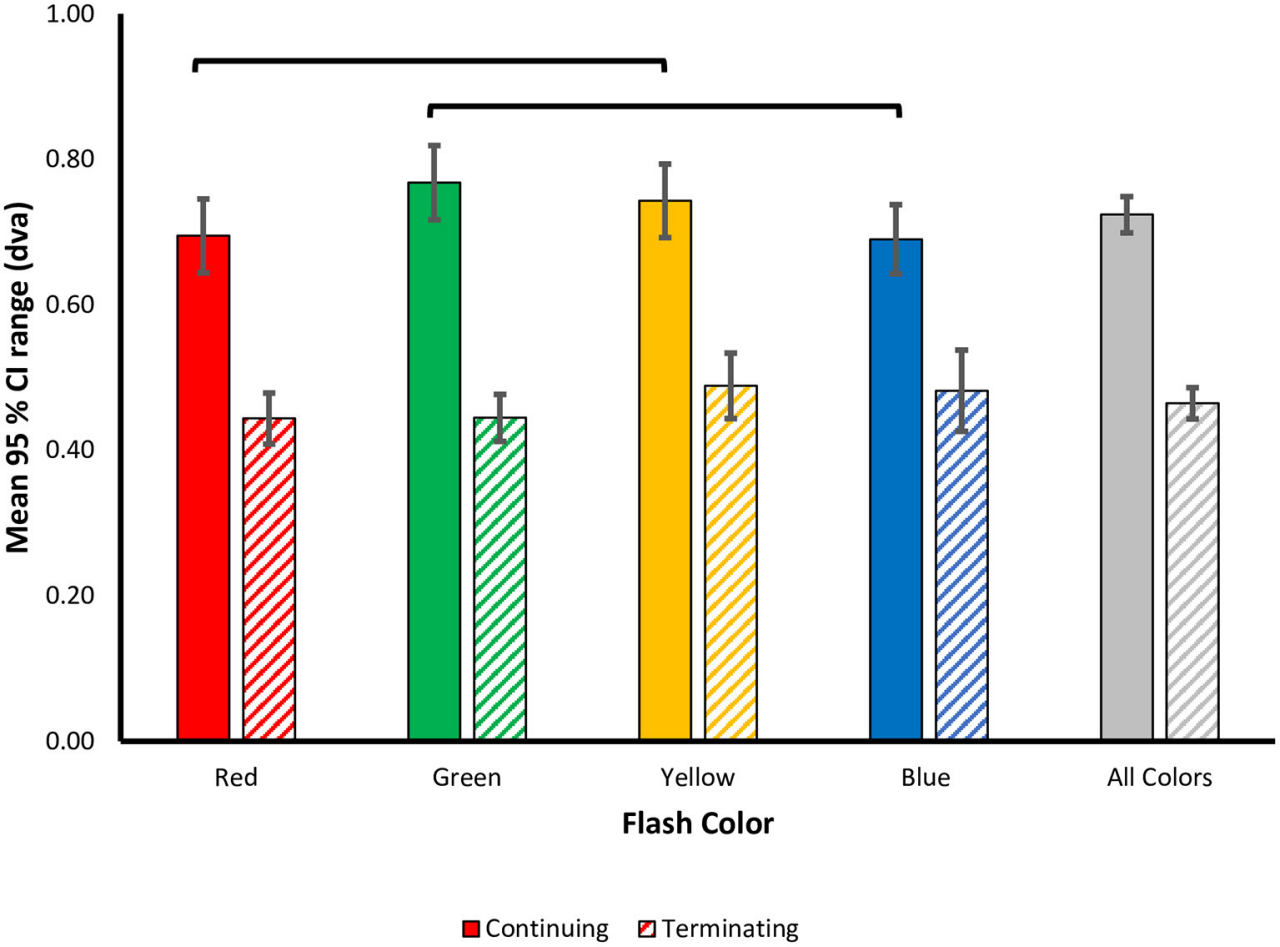
Color modulation of response variability in the flash jump illusion. Precision in responses was measured as the mean 95% CI range ± SEM and plotted as a function of flash color for each motion path. Lines represent color differences unadjusted for multiple comparisons.

To examine the effect of flash color on the variability of responses, i.e., participants' precision in localizing the flash, mean 95% CI ranges were calculated for each flash color in the terminating and continuing conditions (Figure 5). A 4 (Flash Color: red, green, blue, yellow) X 2 (Motion Path: terminating, continuing) repeated measures ANOVA was conducted on this precision metric. There was a significant main effect of motion path [*F*_(1, 69)_ = 89.269, *p* < 0.001, $\eta_{p}^{2}$ = 0.795], with larger 95% CI ranges for the continuing [*M (SD)* = 0.72 (0.24)] compared to the terminating [*M (SD)* = 0.46 (0.21)] motion condition, suggesting more precise representation of the flash location in the terminating condition. Unlike in the perceptual shifts, there was no statistically significant differences in mean 95% CI ranges based on flash color [*F*_(3, 69)_ = 2.283, *p* = 0.087, $\eta_{p}^{2}$ = 0.090] or a statistically significant interaction between motion path and flash color [*F*_(3, 69)_ = 2.256, *p* = 0.09, $\eta_{p}^{2}=$ 0.089].

As with the perceptual shift analysis, we next carried out separate one-way ANOVAs for each motion path. In the continuing path condition, there was a significant main effect of flash color [*F*_(3, 69)_ = 2.879, *p* = 0.042, $\eta_{p}^{2}=$0.111], with less response variability for red [*M (SD)* = 0.695 (0.25)] and blue [*M (SD)* = 0.690 (0.23)] flashes, vs. more response variability for green [*M (SD)* = 0.77 (0.25)] and yellow [*M (SD)* = 0.74 (0.25)] flashes. Qualitatively, the pattern of color effects matches the pattern observed in the perceptual shifts, wherein, red and blue flashes resulted in more precise representations of the flash location, compared to green and yellow. This pattern of results was robust across individuals as 17 out of 24 participants produced more precise estimations of flash location for red compared to green flashes, and 18 out of 24 participants produced more precise estimations for blue compared to yellow flashes. *Post-hoc* tests with Benjamini-Hochberg corrections for multiple comparisons revealed no significant difference in 95% CI ranges for all flash colors (all corrected *p* ≥ 0.054). Given the significant main effect of flash color on response precision, how the pattern qualitatively matches the perceptual shifts, and the higher variability in the precision metric itself, uncorrected pairwise comparisons were also conducted to discern the role of statistical power. We found a significant difference in 95% CI ranges between red and yellow (uncorrected *p* = 0.043) and green and blue flashes (uncorrected *p* = 0.009), while all other contrasts were not significant (uncorrected *p* > 0.05), supporting the qualitative pattern of color modulations (Figure 5).

For the terminating motion path, the main effect for flash color on response variability was not statistically significant [*F*_(3, 69)_ = 1.481, *p* = 0.227, $\eta_{p}^{2}$ = 0.061]. We conducted planned pairwise comparisons finding no significant difference between the mean 95% CI ranges for all color contrasts (corrected *p*'s > 0.05) (Figure 5). Similar to the perceptual shift results, the pattern of color modulations on response variability differed between the two motion paths.

## Discussion

We found a larger shift in the flash mislocalization in the continuing compared to the terminating condition, consistent with a prior study of the flash jump effect (Sundberg et al., 2006). These findings are consistent with other studies of motion-induced position shifts such as the related flash-lag illusion, where the effect is not observed or reduced in the flash-terminated condition, but persists if the flash occurred at the beginning or during the motion sequence (Khurana and Nijhawan, 1995; Eagleman and Sejnowski, 2000; Kanai et al., 2004; Nijhawan et al., 2004; Watanabe, 2004). The absence of motion cues after the flash in the terminating condition allows for a more veridical prediction of flash position as there are no subsequent bars. Previously reported color modulations of cognitive functions have suggested that color can intrinsically modulate attentional and cognitive processes in the absence of a task demand (Lindsey et al., 2010; Tchernikov and Fallah, 2010; Fortier-Gauthier et al., 2013; Pomerleau et al., 2014; Blizzard et al., 2017). In the current study, we sought to investigate whether such color-dependent modulations are dependent on Bayesian perceptual processes in the visual system or whether these color modulations are a result of associative learning for specific higher-order executive functions only. We hypothesized that if different colors had different weights in terms of Bayesian priors, then color would modulate the strength of the flash jump effect.

Consistent with this hypothesis, the color of the flash had a significant impact on its perceived location. Participants demonstrated both improved accuracy (i.e., more veridical reports of the flash location) and improved precision (i.e., less variability in responses) in localizing the flash when it was red or blue compared to green and yellow. Comparing across the colors, the smaller the mislocalization the greater the precision in determining where the flash occurred, suggesting that the color of the flash modulated the strength of its representation in the visual system. This proposed advantage in representational strength is consistent with the advantage found for red or blue pop-out targets in visual search (Lindsey et al., 2010). There was also a slight advantage of terminating red flashes over other colors such as blue and green, as participants were more veridical in reporting their location as well. This small improvement in accuracy for red flashes in the terminating condition may arise from a stronger weight for red signals in stopping, as has been shown in studies investigating response inhibition (Blizzard et al., 2017; Ghasemian et al., 2021). The lack of significant differences between the other colors in the terminated condition may be due to the smaller magnitude of the perceptual shifts when the flash terminates the motion sequence. Overall, red and blue flashes in the continuing motion condition produce more precise and veridical representations of the flash location than green or yellow, which is the focus of subsequent discussion sections.

### Underlying Mechanism for Color Modulation Effects

Next, we consider various mechanisms that could give rise to the color modulation of the flash jump effect. Tchernikov and Fallah (2010) described an intrinsic color hierarchy, where when participants automatically pursued one of two superimposed surfaces differing in isoluminant color, the color determined which surface was selected and the speed of the smooth pursuit. For target selection, the strength of the colors is greatest with red, followed by green, yellow, and blue, while pursuit speed was based on the distance between the two colors in color space, a property of the color representation in area V4 (Li et al., 2014). As the flash jump illusion is also reflected in the responses of area V4 neurons (Sundberg et al., 2006), it would not be surprising to find that the color hierarchy determines the perceptual shift and precision of localizing the flash in the flash jump illusion. However, participants in the current study showed a different pattern than the color hierarchy, as their perceptual shifts were smaller for both red and blue than for green and yellow continuing flashes. Therefore, the color hierarchy does not reflect the differential strengths of the colors when integrated with motion in the flash jump illusion.

As both red and blue produce more veridical and precise localization of the flash over green and yellow, this may instead reflect opponent-process theory (Hering, 1964), based on opponent color channels; red vs. green and blue vs. yellow, where each color of the pair inhibits the other. However, color opponency does not inherently predict which color of each pair would be the stronger. The results of this study are consistent with opponent-process theory, and further suggest that red is dominant over green and blue over yellow. Consistent with this finding, the advantage for red over green has previously been found for response inhibition in the stop-signal task (Blizzard et al., 2017), however green and yellow were not tested. It is interesting to note that the ratio in perceptual shifts between red and green (0.86) is qualitatively similar to the difference between blue and yellow (0.83) suggesting that each opponent pair may be weighted similarly. However, future studies will be necessary to fully understand the relative weightings of the colors in each opponent pair. Since V4 is the last stage that receives color opponency information (Conway, 2009), this input is likely modulating the color-selective neuronal responses giving rise to the perceptual shift in the flash jump illusion.

### Motion-Induced Position Shifts

The flash jump illusion is one of many phenomena related to motion-induced position shifts; other related examples include the flash-lag (Nijhawan, 1994; Whitney and Murakami, 1998; Eagleman and Sejnowski, 2007; Khoei et al., 2017), flash-grab (Sinico et al., 2009; Cavanagh and Anstis, 2013), flash-drag effects (Whitney and Cavanagh, 2000; Murai and Murakami, 2016). Multiple explanations have been proposed to explain such motion-based mislocalization errors, including the differential latency, discrete sampling, motion biasing, and motion extrapolation hypotheses; for a review of prominent theories (see, Nijhawan, 2002; Hubbard, 2014). The results of the current study provide an opportunity to distinguish between these hypotheses, supporting those that could integrate color modulation effects and refuting those that could not.

The differential latency hypothesis proposed by Whitney and Murakami (1998) posits that moving targets are processed faster, with shorter neural delays, than stationary flashed targets (Cai and Schlag, 2001; Jancke et al., 2004; Subramaniyan et al., 2018). In consideration of the *flash-lag* effect, by the time the static flash is perceived, the moving bar is perceived to be further along its trajectory. Previous studies have reported temporal processing differences between color and motion systems (Moutoussis and Zeki, 1997; Viviani and Aymoz, 2001). However, for the differential latency hypothesis to explain color modulation of the *flash- jump* effect, the latency difference between color and motion would need to vary depending on the color of the flash, which would require that different colors be processed with different latencies. Indirect evidence contrary to this account comes from Blizzard et al. (2017), where it was demonstrated that reaction times were not differentially modulated based on the color of the go signal, both when the color was task-irrelevant or color was used as the discriminator for response selection. The range of mislocalization differences between red/blue and green/yellow flashes is 0.2–0.24°, which corresponds to latency differences between the colors of ~17–20 ms. While neurophysiological approaches have revealed no differences of that magnitude in neuronal latencies for different colors in area V4 (e.g., Chang et al., 2014), future neurophysiological studies would need to precisely measure color latencies in multiple areas along the ventral visual stream to further test the differential latency hypothesis.

More recently, Schneider (2018), proposed the discrete sampling hypothesis which posits that the visual system samples input into discrete moments or time windows, each with a duration, D. This discrete sampling hypothesis suggests that, during the flash-jump illusion, the color change occurs at a point in time during one moment or sampling window; however, the location of the moving target is recorded as its final position within that time window. By this hypothesis, the color flash would be displaced by D/2 from its actual time of onset. The discrete sampling hypothesis is a modification of the differential latency theory, as both suggest temporal processing differences for color and motion information. However, the discrete sampling hypothesis compartmentalizes color and motion information into discrete moments based on these latency differences. If the latency difference between two events (i.e., color change and motion) is small, they are registered in the same moment, thus perceived together, but, if there is a large latency difference between color and motion, then these two events are registered into different moments, leading to the flash jump effect. Based on the speed of the apparent motion sequence, the average flash jump effect of 1.33° corresponds to a latency difference of ~111 ms, which would be consistent with discrete sampling. However, the discrete sampling hypothesis does not currently predict differences in perceived position shifts based on flash color, as the sampling differs between modalities, rather than between individual colors. For this theory to explain color-dependent modulation of the perceived shift in flash location, as the duration of each perceptual moment would not vary based on the color of the flash, there would need to be latency differences between the different color flashes that would shift green and yellow into a later discrete moment than red and blue. While there is no current evidence for varying latencies for the different colors (Chang et al., 2014; Blizzard et al., 2017), if future neurophysiological studies find latency differences in color opponent areas, and those differences are large enough to cause different color flashes to fall into different moments, then the discrete sampling hypothesis could potentially account for the color dependent modulation of the flash-jump effect.

Temporal integration theories of motion-induced position shifts in the flash-lag illusion (Brenner and Smeets, 2000; Eagleman and Sejnowski, 2000) suggest that position estimates are computed based on information that is collected over a period of time after the flash. This post-diction or motion-biasing hypothesis postulates that the flash triggers the start of the motion integration window where information about the position of a moving target is collected for another ~60 ms (Brenner and Smeets, 2000) to ~80 ms (Eagleman and Sejnowski, 2000, 2007) after the flash. Therefore, the final position of the colored flash is shifted along the trajectory of motion by signals that arrive in the next ~60–80 ms. For this hypothesis to account for color-dependent modulations of the flash jump effect, different colors would need to either initiate the start of the integration window at different times or produce different integration window durations. Given that colors have been shown to modulate attentional resources (Tchernikov and Fallah, 2010), the attentional strengths of different colors could similarly modulate the integration window. In terms of the attentional color hierarchy, red is the strongest and blue is the weakest of the four colors tested (Tchernikov and Fallah, 2010). A color-dependent attentional modulation of the integration window would then predict less of a shift for red than green or yellow, as was found in this study, but also that blue would produce the largest shift. In contrast, the current study showed that blue (as well as red) produced smaller shifts than green and yellow. Therefore, the incorporation of the attentional color hierarchy into the postdiction hypothesis cannot explain the current results.

The motion extrapolation account posits that the perceived position of a moving target is extrapolated forward along its trajectory, based on its previous history, to compensate for neural delays in processing (Nijhawan, 1994). More recently, Bayesian frameworks for object localization have been used to explain motion extrapolation (Lisi and Cavanagh, 2015; Khoei et al., 2017; Hui et al., 2020). For example, Lisi and Cavanagh (2015) proposed that the previously perceived location of a moving object becomes a Bayesian prior for the estimate of its upcoming motion. Similarly, the parodiction hypothesis proposes that the visual system predicts an object's final position as its most probable position (Khoei et al., 2017). These are both Bayesian models, which integrate sensory information with an internal a priori distribution to give a probability distribution function of the object's position. Next, we will describe how extensions of these Bayesian models provide a framework by which color can modulate the degree of the flash-jump effect.

### Bayesian Framework

Bayesian models of perception suggest that if the input is sparse, variable, or noisy (visual or perceptual noise), the visual system makes a prediction by biasing its perception toward typical objects or representations based on priors (Knill and Richards, 1996; Rao et al., 2002; Kersten and Yuille, 2003; Kersten et al., 2004; Feldman, 2012). As there is already work supporting Bayesian frameworks for motion processing (Jacobs, 1999; Weiss et al., 2002; Lisi and Cavanagh, 2015, Gershman et al., 2016; Hui et al., 2020; Yang et al., 2021), Sundberg et al. (2006) proposed a similar Bayesian approach for the estimation of flash location relative to bar locations in the flash-jump paradigm. The authors observed a retinotopic shift in color-selective V4 neurons for both the terminating and continuing conditions and proposed that this retinotopic shift in V4 cells provides a physiological basis for our perception of the flash- jump effect. Although a retinotopic shift was observed for both conditions, perceptually, human observers do not report large mislocalizations in the terminating condition. Therefore, to explain this discrepancy, Sundberg et al. (2006) proposed a Bayesian model of sensory integration, wherein the shifted representation of the flash (V4 responses) is integrated with a representation of all presented bar positions (likely occurring in a later stage color-insensitive area), giving rise to a joint probability estimate of the flash relative to actual bar locations. In the continuing condition, color-insensitive areas maintain the location of all bars in the sequence, since all locations were presented, therefore, the resultant joint probability function represents the shifted position signaled by the mislocalization in the V4 neurons. However, in the terminating condition, bars beyond the flash are not presented, therefore there are no representations for any further bar positions in color-insensitive areas. When the shifted flash representation from area V4 is integrated with this representation of the terminated condition bar locations, the resultant joint probability estimate is restricted to the veridical location, resulting in the absence of a perceptual shift in the terminating condition.

We propose a modification of this Bayesian framework to explain how the representation of the flash in the color selective area (e.g., area V4) is mislocalized and how color modulates the magnitude of this shift. We propose that the V4 mislocalization reported by Sundberg et al. (2006) is produced by an earlier Bayesian framework for feature integration, where the motion extrapolation signal is combined with the color signal that is weighted differently depending on the color priors.

**Figure 6:**
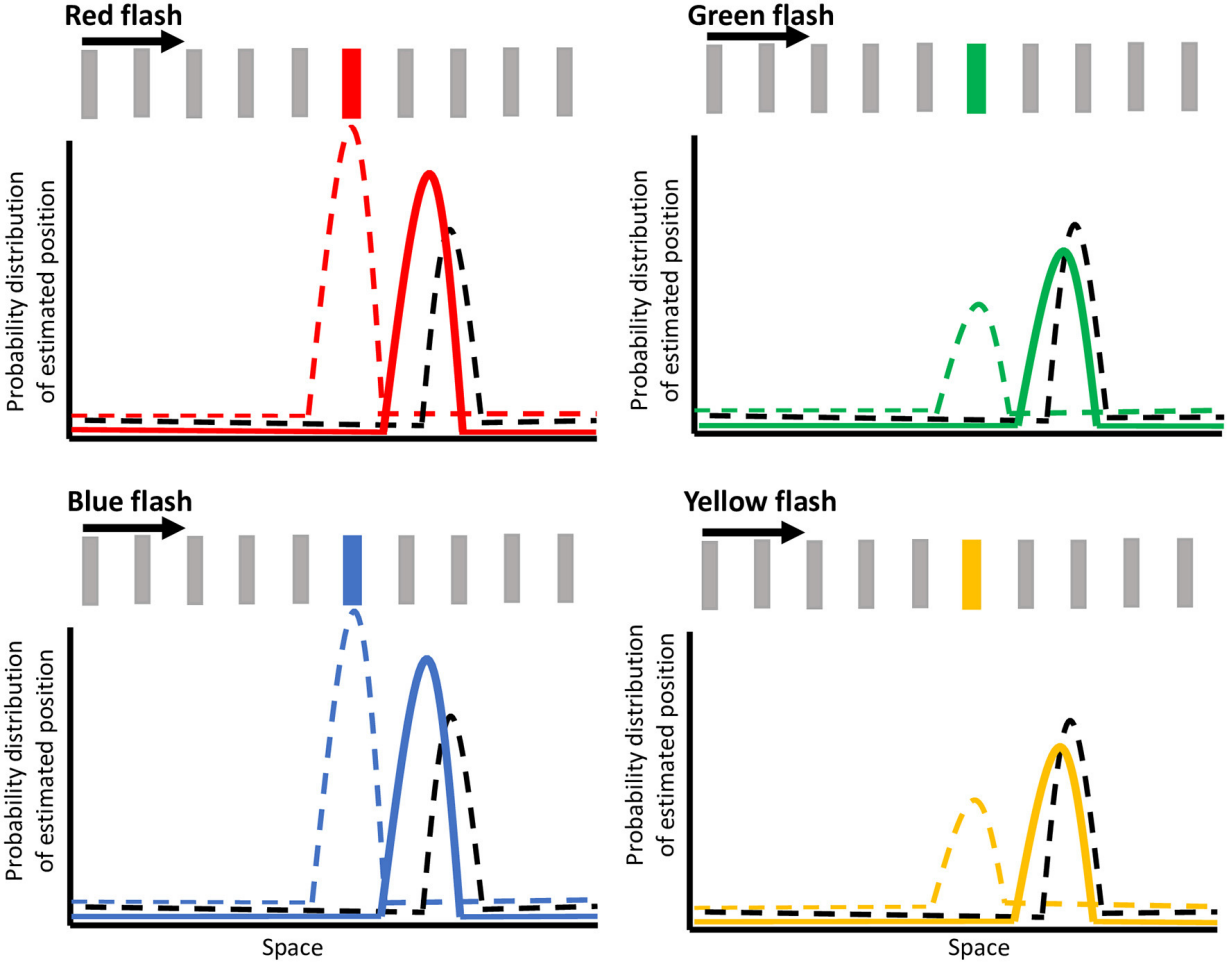
Illustration of the Bayesian model of color modulation. The dashed black curve depicts the motion extrapolation-dependent position representation for the moving bar at the onset of the flash. The dashed colored curve depicts the estimation of the flash location at the onset of the flash for red, green, blue, and yellow flashes, with the height representing the weight of that estimation. The integration of these two signals is depicted by the solid-colored curve, which represents the probability density function for the mislocalization of the flash, as represented by V4 neuronal responses. Note that this illustration depicts the flash-continuing condition with the bar moving in the rightward direction and the spacing between bars has been exaggerated for illustration purposes.

Figure 6 shows hypothetical probability distributions for the flash locations in the continuing condition for red, green, blue, and yellow flashes. The dashed black curve illustrates the estimate for motion position at the onset of the flash, arising from a motion selective area such as MT. Note that the estimate for motion position is further ahead along the direction of motion at the time of the flash due to the motion extrapolation prior (Nijhawan, 1994; Sundberg et al., 2006; Lisi and Cavanagh, 2015; Khoei et al., 2017; Hui et al., 2020). Anterograde and retrograde tracer studies have confirmed the presence of bidirectional connections between area MT and V4 (Ungerleider and Desimone, 1986; Ungerleider et al., 2008). This motion information feeding into V4 has also been shown to modify the selectivity and responses of V4 neurons (Tolias et al., 2005). Therefore, we suggest that the mislocalization in V4 responses (Sundberg et al., 2006) arises from the integration of incoming motion signals with incoming weighted color opponency information. At flash onset, a representation of the bar's motion (from motion selective areas) which is shifted forward due to motion extrapolation (black dashed curve in Figure 6) is integrated with a representation of the color flash (from color opponent cells) at its veridical location (colored dashed curve in Figure 6), giving rise to a posterior probability distribution (solid colored curve in Figure 6) that is shifted forward. The color-dependent modulation of the flash jump effect is dependent on the representation of the flash (color dashed curve) being weighted differently by color. The dashed colored curve illustrates the estimation of the flash location (i.e., color selective area) at the onset of the flash for each of the red, green, blue, or yellow flashes, with the height of the curve representing the weighting of that representation. Based on our results, a higher weighting is given for the stronger colors (red and blue) than the weaker ones (yellow and green). The difference in weighted priors for these colors are likely due to color opponency input, wherein red in red vs. green, and blue in blue vs. yellow are the stronger colors of the pairs, even at isoluminance. The resultant probability density function (depicted as solid colored curves in Figure 6) is less shifted forward for red and blue resulting in a predicted flash location closer to its veridical location, but is more shifted for yellow and green, resulting in a larger mislocalization or jump of the flash. The integration of the motion and color opponency signals thus produces the mislocalized responses in area V4 neurons, meaning that the integrated probability distribution functions from our model (solid-colored curves) correspond to the shifted V4 color signal in the model proposed by Sundberg et al. (black curves in Figure 4 of Sundberg et al., 2006). As Sundberg and colleagues proposed, this shifted V4 signal is then integrated with actual bar locations (represented in a later stage color-insensitive area) in a second Bayesian process to give an estimate of flash location relative to the continuing or terminated conditions. As we observed small mislocalizations in the terminated condition as well, we propose that similar to the continuing condition, the representation for the final bar position in the terminated condition would also be slightly shifted forward due to motion extrapolation (Lisi and Cavanagh, 2015; Khoei et al., 2017). Then, when the color weighted flash information is integrated with this motion estimate, the probability density function would be color modulated around a slightly shifted mislocalization, consistent with our perceptual results. Since the flash-jump mislocalization is found in the responses of area V4 neurons (Sundberg et al., 2006), this proposed mechanism predicts that color modulation would also be reflected in area V4, where the mislocalization would vary based on the color of the flash, although further studies would be needed to confirm it. More generally, these results provide further support that perception and feature integration follow a Bayesian framework.

## Conclusion

These results show that the color of the flash modulates its perceived location in the flash jump illusion, affecting both accuracy and precision metrics. Specifically, red and blue flashes in the continuing motion path are localized both more precisely and with less of an illusory shift than green and yellow, likely based on color opponent mechanisms which provide input into area V4, an area previously shown to encode the mislocalized position of the flash. We propose a Bayesian framework that integrates color opponency priors and motion extrapolation priors to give rise to the flash jump illusion. The color of the flash biases the weight of the priors; resulting in different levels of mislocalizations based on inherent strengths of different colors, where red and blue have stronger representations than green and yellow. This is further support for perception and feature integration being dependent on Bayesian mechanisms.

## Data Availability Statement

The raw data supporting the conclusions of this article will be made available by the authors, without undue reservation.

## Ethics Statement

The studies involving human participants were reviewed and approved by York University's Human Participants Research Committee. The patients/participants provided their written informed consent to participate in this study.

## Author Contributions

HS wrote the manuscript, collected, and analyzed the data. HJ designed the experiment and data analysis, programmed the experiment, and revised the manuscript. MF designed the experiment, data analysis, and revised the manuscript. All authors contributed to the article and approved the submitted version.

## Conflict of Interest

The authors declare that the research was conducted in the absence of any commercial or financial relationships that could be construed as a potential conflict of interest.
